# “This is an opportunity for leadership to lead, but leadership has disappeared”: A qualitative case study of clinical and translational scientists during COVID-19

**DOI:** 10.21203/rs.3.rs-310774/v1

**Published:** 2021-03-16

**Authors:** Chelsea Leonard, Brigid Connelly, Karen Albright, Heather Gilmartin

**Affiliations:** VHA Eastern Colorado Healthcare System; VHA Eastern Colorado Healthcare System; VHA Eastern Colorado Healthcare System; VHA Eastern Colorado Healthcare System

**Keywords:** Leadership, COVID-19, Clinical and Translational Science, Qualitative, Colorado

## Abstract

In March of 2020, academic research centers in Colorado were closed to prevent the spread of COVID-19. Scientists and research staff were required to continue their work remotely with little time to prepare for the transition. This survey study used an explanatory sequential mixed method design to explore clinical and translational researcher and staff experiences of the transition to remote work during the first six weeks of the pandemic. Participants indicated the level of interference with their research and shared their experiences of remote work, how they were impacted, how they were adapting and coping, and any short or long-term concerns. Most participants indicated that remote work interfered with their research to a moderate or great degree. Participant stories illuminated the differences of remote work prior to and during COVID-19. They described both challenges and silver linings. Here we describe three themes that highlight the challenges of transitioning to remote work during a pandemic: 1) Leadership: *“This is an opportunity for leadership to lead, but leadership has disappeared”;* 2) Parenting: *Parents are “multitasked to death” every day,* and 3) Mental health: *The COVID-19 experience is “psychologically taxing”;The* study findings can be used to assist academic, hospital, department, and team leaders in building community, resiliency, and support productivity during current and future crises.

## Background

The COVID-19 pandemic has caused far reaching challenges to the health, safety, and livelihood of citizens worldwide. In the United States, federal and local governments declared health emergencies to limit the spread of the COVID-19 virus (Gostin & Wiley, 2020). In March of 2020, the Governor of Colorado implemented a mandatory statewide stay-at-home order (Moreland et al., 2020; Polis, 2020). Across Colorado, offices, schools, laboratories, and businesses were closed and citizens were instructed to stay-at-home except for crucial activities (Polis, 2020). For the first time, Colorado-based clinical and translational scientists and research staff were asked to continue their research from home. Those in leadership positions were asked to support researchers and staff who had minimal experience with remote work and were dealing with the personal, family and societal stressors of living and working during a pandemic. With no time to prepare, researchers and staff rushed to create home offices while leaders looked to each other and to national organizations for guidance. During the early weeks of the COVID-19 pandemic, there were concerns that social distancing would delay vital scientific research (Impey, 2020), as leaders and staff struggled to adapt to remote work (Lee, 2020).

Remote work is common in many professions, but not standard practice for clinical and translational scientists and staff. This is because clinical scientists work directly with human subjects or on materials of human origin, such as tissues, specimen, and cognitive phenomena Translational scientists apply discoveries generated during laboratory research and pre-clinical studies to the development of trials and studies in humans. The existing remote work literature primarily focuses on employees who volunteer to work outside of the office setting (Nakrošienė, Bučiūnienė, & Goštautaitė, 2019). This group seeks remote work opportunities to increase work-life flexibility (Kelliher & Anderson, 2010), reduce commute times (Kelliher & Anderson, 2010), and reduce office distractions (Choudhury, Foroughi, & Larson, 2020).

Though many benefits are reported, voluntary remote workers also report increased feelings of social and professional isolation, missed informal learning opportunities, and decreased support from leaders, peers and institutions (Kelliher & Anderson, 2010). One takeaway from this body of literature is that leaders must assume more responsibility for working with staff who are at a distance (Neufeld, Wan, & Fang, 2010).

For clinical and translational scientists and staff newly working from home during COVID-19, establishing new methods of leading, managing, communicating, collaborating, and working was critical. Simultaneously, these scientists and staff learned how to share a workspace with roommates or family while navigating the uncertainty of the pandemic, and providing social, emotional, and homeschooling support to children (Primack & Setash, 2020). To ensure vital scientific research continues for the duration the COVID-19 pandemic, it is essential to explore how this novice group of remote workers is adapting, coping and being supported by leadership. The objective of this paper is to describe experiences of Colorado clinical and translational scientists and staff and understand how leadership can foster remote work and collaboration among teams of scientists.

## Methods

### Design and Setting

This study employed an explanatory sequential mixed methods research design (quantitative à QUALITATIVE = explanation), which included a quantitative survey with qualitative open text items (Tashakkori, Johnson, & Teddlie, 2020). The qualitative results built better understanding of the quantitative survey findings. Due to the time sensitive nature of this work, the survey used a convenience sample consisting of members of the Colorado Clinical and Translational Sciences Institute (CCTSI). The survey methods have been previously published (Gilmartin, Connelly, Hebbe, Battaglia, & Kwan, 2021). In brief, the CCTSI is a Clinical Translational Sciences Award site funded by the National Center for Advancing Translational Sciences to provide resources to support basic, translational and clinical researchers to move scientific discoveries to clinical innovations that diagnose, prevent, or treat disease (Sokol, 2020). Based at the University of Colorado Anschutz Medical Campus (UC-AMC), the CCTSI partners with multiple hospitals and academic institutions. Survey invitations were sent to current CCTSI members (e.g., faculty and staff of the multiple partner institutions) over the age of 18 years old (n=5,067). The survey opened April 28, 2020 and was available until May 11,2020.

### Remote Work Survey

Survey questions were drawn from the remote work literature (Felstead & Henseke, 2017; Kahana, 2020; Staples, 2001), experiences posted to Twitter (#remote work; #WFH) in the first weeks of COVID-19, and the authors’ personal experiences with remote work. The following demographics were captured in the survey: Respondent’s age, professional credentials, gender, faculty investigator level, CCTSI partner site, and prior remote work experience. Participants rated the extent to which remote work during COVID-19 interfered with their research activities (i.e., does not interfere, interferes somewhat, interferes to a great extent). The survey included an optional open text prompt asking respondents to: *“Please share how you are doing during the COVID-19 pandemic, in your story, please consider including information on: How remote work during the COVID-19 pandemic has impacted you; How you are adapting; How you are coping; Your concerns in the short and long term.”*

### Statistical and Qualitative Analyses

Survey data were exported from RedCap to SPSS (IBM, version 27) for descriptive analyses of the quantitative data and to ATLAS.ti Scientific Software Development GmbH for the qualitative data. The data were stratified by the extent remote work during COVID-19 interfered with research activities. Responses to the open-ended question were analyzed using iterative, team based inductive-deductive content analysis to identify themes (Bradley, Curry, & Devers, 2007; Patton, 2014). Initial code categories were based on factors related to work structures (e.g. leadership, communication, workspace and infrastructure), expressions of emotions, and facilitators/barriers to working remotely. Inductive codes were used to identify emergent ideas and were added throughout coding. Consensus was reached using a team-based approach (Bradley et al., 2007). Three analysts (CL, BC, and HG) group coded twenty percent of responses and discussed points of disagreement. Analysts met weekly to discuss their own expectations of the data and record any biases they might have due to their own remote work experience (Patton, 2014). Analyses continued with emergent themes, with attention to differences between groups who reported none, somewhat, and great interference with their research. The study was deemed non-human subjects research by the Colorado Multiple Institutional Review Board (20–0892).

## Results

Of the 5,098 current CCTSI members, 322 responded to the survey and 260 completed the open text item. Most participants were female (n=198, 76%), 21 to 73 years old (mean=42 years), with a PhD (n=117, 46%) or MD (n=42, 55%). The most frequent research role reported was faculty investigators (n=135; 52%) at the Assistant Professor level (n=52; 39%), followed by research clinical staff (n=82; 32%). Very few respondents reported a history of remote work; 51% (n=133) had never worked remotely and 27% (n=69) reported working remotely only once a week prior to the pandemic. Only 11% (n=28) had previously worked from home > 2 days a week. Remote work during COVID-19 was reported by 44% (n=113) of participants to somewhat interfere with their research. For 33% (n=85), remote work interfered to a great extent (Table 1).

### Qualitative Findings

Participants stories illuminated the difference between working from home prior to COVID-19 and working from home during COVID-19. A female research clinical staff summarized it as, *“We are not working from home rather working through a pandemic.”* Despite the challenges they faced, many participants described silver linings including interest in continuing to work from home after the pandemic ends. We identified three main themes related to the CCTSI members experiences when transitioning to remote work during the first weeks of COVID-19: leadership, parenting, and mental health.

### Leadership: *“This is an opportunity for leadership to lead, but leadership has disappeared”*

Many participants described concerns over their university, department, and team leadership response to COVID-19. Many who experienced great interference with their research voiced disappointment with university and department leadership’s lack of engagement. A male, non-faculty investigator voiced the concern of many, *“This is an opportunity for leadership to lead, but leadership has disappeared”* Some shared that leaders had not acknowledged the difficulties they were experiencing or provided clear plans for how to continue working from home. For example, a faculty investigator shared her frustrations, *“I feel this situation was presented as “temporary” and that we were in crisis mode. However, now that we’re 6 weeks in and there’s no clear end in sight -* I *would appreciate if leadership would reflect that and start providing plans.”* Another female faculty investigator mentioned that unclear communication from university leadership caused unnecessary stress and anxiety, *“Having the Chancellor’s email come out and then the more detailed plan emailed a day later created panic, confusion and fear.”*

Many CCTSI partner sites instituted hiring freezes during this time. Participants were frustrated with this blanket policy and how it impacted their work. A female non-faculty investigator shared, “A major concern is that we are not allowed to hire more people to conduct our program’s research.... It is very frustrating that while we have grant funds for personnel, we are not able to use them.” Another female faculty investigator wrote there was “NO REASON’ for the hiring freeze, “I bring in millions of grant funding, provide hundreds of thousands of indirects... these decisions are going to hurt the many investigators like myself...”

Communication from leadership specific to research activities, career and personal health were viewed as absent by some or confusing by others. A female faculty investigator shared, “There’s been no communication acknowledging the impact that this situation has on those of us that are dependent on human subject data or what the plan may be moving forward” Another female faculty investigator indicated, “I had to stop working on my grant because my teaching workload tripled to switch my class online with a very short notice and help. Not sure what to expect from the leadership regarding this and how promotions will be managed” A female research clinical staff commented on frustrations with lack of communication from her mentors, “Without any communication from mentors, I am only guessing at the expectations and goals.”

Many participants described mixed messages from university and department leadership. The times were acknowledged as challenging, but researchers were also encouraged to maintain productivity. A female faculty investigator shared; *“It often feels like leadership is simultaneously telling us to take care of ourselves and our families but also emphasizing how much productivity matters.”* I n some cases, there was no communication from leadership, which was perceived as inappropriate. A research clinical staff shared *“Was doing fine until furloughed.* I *understand the need, but not to receive a personal phone call from department chair to show concern or express confidence shows lack of concern and respect for employees.”*

Examples of leadership support came from those who reported minimal research interference. A research clinical staff member shared, “My principal investigator is incredibly helpful and encouraging, has come up with new ways and systems to facilitate productivity, regularity, and consistency in our team...” A faculty investigator noted, “My supervisor has been very understanding and flexible with when I get my work done...” and a research administrator expressed, “I truly feel that my work is valued by my bosses.”

### Parenting: *Parents are being “multitasked to death” every day.*

Participants with children described challenges working from home while parenting. Attempts to maintain productivity and work-life balance was exhausting for many, as expressed by a female faculty investigator, “Society thrust new responsibilities on parents with remote learning. This means we are multitasked to death every day.” Another female faculty investigator shared, “I am caring for a young daughter - any time that is not in structured meetings or telehealth encounters is spent caring for her.” A male faculty investigator echoed this experience, “Overall, I have been much less productive than if I was not having to balance childcare with getting work done.”

Participants with children also discussed how they were impacted by the closure of day care centers, schools, sports, and child activity programs. A female faculty investigator explained, “The pandemic has... taken a way many of the things that allowed me to work previously: loss of school and activities for kids....” Another faculty investigator described wearing many hats, “...I am in mom-mode 24/7now. I spend my days bouncing between being a full-time chef housekeeper, 7th grade teacher, 5th grade teacher, classroom manager and school counselor.”

Remote learning was a significant stressor because schools were not prepared, and many children cannot manage their own learning. As a female faculty investigator shared, “I *have a first grader and* I *am now responsible for her education.* I *feel like every day* I *have to choose between ignoring my work or her school.”* Parents shared that supporting the educational and emotional needs of their children was a priority, but stressful. A female faculty investigator summed it up for many, *“Iam frazzled, constantly distracted and stressed... Every day is a failure of some sort.”* Multiple participants expressed concerns over the long-term loss of childcare options. A female faculty investigator shared, *“The thought of kids at home all fall too strikes fear. How long can we work a job that takes more than full time anyways AND home school 3 other people.”*

The differential burden of parenting on job types and genders was a frequent topic. A female faculty investigator explained, “My spouse is also in healthcare, but because I do research and have more flexibility for where I can work, the childcare responsibility and schooling falls on me.” Another female faculty investigator reflected, “COVID has exacerbated the usual ‘second shift’ imbalances on me as the wife in a two academic physician household...” The professional impact on women working full-time and parenting full-time was a concern. A female faculty investigator shared, “In the longer term, I am concerned that I won’t be able to publish as much as my colleagues, that I won’t be able to compete for research grant funding that my colleagues are applying for...”. To maintain some productivity while parenting, many reported working long hours, as described by a female faculty investigator, “... I’m losing sleep staying up late to get any work done that might require more focus and attention than I can give during the day. I haven’t gone to sleep before midnight in weeks...”

### Mental Health: *The COVID-19 Experience is “Psychologically Taxing”*

Almost all participants who expressed negative impacts on their mental health also reported some or great interference with their research. The pandemic and working from home generated anxiety about their own health and safety, along with concerns for family and colleagues. A female faculty investigator shared, “I have found the whole COVID experience to be extremely psychologically taxing as I worry about the health of my family.” A female research clinical staff expressed, “I am worried about my coworkers inside and outside the hospital and how they are dealing with things.” The stay-at-home restrictions were challenging for those who live alone, “I live alone and was fairly isolated before the pandemic, so not being able to go to work and interact with co workers and study subjects as normal has been very difficult. My mental health is definitely suffering. [...].

The many unknowns surrounding the COVID-19 pandemic were a significant stressor, as described by a female research clinical staff, “I was unable to get significant amounts of work done for at least the first 2–3 weeks of work from home because my mind could not settle on the work because my non-work life was filled with uncertainty, stress, and fear.” While a female faculty investigator described: “There are good days and bad days, and no way to predict which one is coming next [...] It is difficult to lead a group when there are so many unknowns.”

Notably, some participants reported improved mental health during remote work. These participants were also those who reported no interference with their research. Many in this group reported decreased stress, as noted by a research clinical staff, “I have actually felt less stressed and happier since I’ve been working at home.” While a female faculty investigator shared, “I have loved this change for my family [...] No stress of driving into work, don’t feel spent by the end of the day and get to see my family more. I am a happier, more balanced person, despite the worries COVID has introduced into daily life.”

## Discussion

We identified three themes related to remote work during the first six weeks of the pandemic: leadership, parenting, and mental health. There were notable differences in expressions of positive and negative emotions between those who reported no interference with their research and those who reported some or great interference. These differences highlight that for some, the move to remote work during COVID-19 was a bump in the road. For others it was a life-changing, stressful event. Here we discuss our findings and share actions and implications for leaders to build community, resiliency and support productivity during current and future crises.

Remote work in the time of COVID-19 looked and felt different than remote work before the pandemic. Specifically, remote work during COVID-19 was mandatory and occurred during a worldwide crisis. Most existing literature is in the context of occasional remote work that is largely voluntary (Wang, Liu, Qian, & Parker, 2020). As a result, previously perceived benefits did not represent the experience of those who suddenly moved to full-time remote work during COVID-19. While respondents indicated both positive and negative experiences, there was a clear need to understand how to support newly remote workers and address their individual challenges.

Many in our sample described a lack of community, absent leaders, and infrequent and inconsistent communication regarding decisions that impacted their research and their careers. Leadership can promote employees’ sense of belonging, connection and support (Teoh & Kinman, 2020) by allocating time on meetings to check-in with colleagues and recognizing their efforts. Even brief, ad hoc conversations can be helpful (Nembhard, Burns, & Shortell, 2020). Effective crisis management requires effective planning and coordination skills as well as the ability to communicate clear consistent messages in an empathetic manner (McGuire, Cunningham, Reynolds, & Matthews-Smith, 2020). Communication should be frequent, timely and accurate and distributed through mediums used by the target audience (Gittell, 2016). In pre-pandemic remote work, social support was a strong predictor of work success and satisfaction. (Toscano & Zappala, 2020; Wang et al., 2020). Social support was even more critical during COVID-19 due to the social isolation experienced while physical distancing.

Many participants reported anxiety about the pandemic and the impacts on their mental health. There were many sources of anxiety during COVID-19 and no easy solutions (Shanafelt, Ripp, & Trockel, 2020). Leadership can support the wellbeing of employees by actively listening and acknowledging group concerns (Shanafelt et al., 2020). While some groups, such as working parents, faced additional stressors while working remotely during COVID-19, the strategies to support them and build resilience are the same. Leaders can decrease anxiety by being visible and present, openly sharing their personal experiences, and connecting with teams rather than dictating (Hoyt, 2020). Further, leaders can share resources for mental health support that are available within or outside the organization and routinely encourage participation.

Finally, many of our participants discussed concerns around productivity and work-life balance. There was a common perception that remote work during COVID-19 had created “extra time” (Kreeger et al., 2020). However, this did not account for the additional responsibilities (e.g., childcare, homeschooling) and new work (e.g., Zoom meetings)(Fosslien & Duffy, 2020) thrust on remote workers during the pandemic. To alleviate concerns about productivity, leaders can help employees identify priorities, set achievable goals, minimize busy work, and establish high-quality communication channels within the remote workplace (Northrup, 2020). Ensuring remote workers have autonomy in their day-to-day work can help employees manage career and family responsibilities (Gajendran & Harrison, 2007; Kahn & Byosiere, 1992). Work autonomy can also decrease pandemic-related loneliness by increasing proactive behaviors such as initiating or engaging in online interactions and developing creative solutions to problems (Wang et al., 2020). A common productivity strategy we do not recommend is enhanced monitoring. While in some instances monitoring can reduce procrastination, in the pandemic context, excessive monitoring may increase the perceived imbalance between “take care of yourself” while “maintaining productivity” (Wang et al., 2020). Despite the expectation that research continued during COVID-19, many scientists and staff expressed a short-term need to change expectations due to the extreme upheaval caused to society from the pandemic.

### Organizational Leadership Implications

Our data suggest that the COVID-19 pandemic offered a moment to reflect on how healthcare leaders can address the needs of those working remotely in a time of crisis. The literature provides guidance on building great healthcare teams that applies during times of unrest and uncertainty (Traylor, Tannenbaum, Thomas, & Salas, 2020). These include preparing teams to perform by conducting pre-briefing and post-performance huddles that allow groups to strategize together and coordinate actions to complete and then reflect on a project, procedure, or workweek (Salas, Rosen, & King, 2007). These sessions work best when supported by leadership and work well for teams with stable or rotating membership (Christian, Christian, Pearsall, & Long, 2017). To support the mental health of teams, offer organizationally supported psychological or self-care resources. Check in with staff and provide them time to check in with family or friends. This can reduce stress at home and afford brief moments of recovery to build resilience (Alliger, Cerasoli, Tannenbaum, & Vessey, 2015). Finally, we recommend frequent, timely and accurate communication between leaders, teams, and individuals to reduce confusion and stress while ensuring the continuation of the scientific endeavor in times of crises.

### Limitations and Future Directions

The current study is a cross-sectional design and represents a snapshot of participants’ experiences during the first six weeks of the COVID-19 pandemic. Though we conducted rigorous qualitative analysis, it was challenging to disentangle what people were feeling about working remotely versus working remotely during a worldwide pandemic. Further, our convenience sample consisted predominantly of female respondents. Due to this, our findings may not be representative of all CCTSI members, or transferable beyond this sample. The findings must be interpreted within this context. Future studies to understand the long-term experience of CCTSI members living and working during the COVID-19 pandemic are planned. We hope the longitudinal data will highlight essential components of leadership, parenting, and mental health for clinical and translational scientists and staff in the current circumstances and beyond.

## Conclusions

Our findings reflect a growing body of work that acknowledges the challenges of remote work during COVID-19 and adds important perspectives of clinical and translational scientists and staff. Most of our participants were first-time remote workers, and many were caring for children while trying to continue their research. Scientists and staff may require individualized attention from leadership and mentors, including collaborative planning for how they can progress in their careers while taking the unique stressors of the pandemic into account. Further, our findings suggest that clear and consistent messaging from leadership, as well as empathy and presence, are critical to maintain morale and mental health. Finally, the structures built during the COVID-19 pandemic can continue to foster remote work when the pandemic ends. Many participants discussed the personal and professional benefits of remote work and wished to continue working remotely to some degree.

## Figures and Tables

**Table 1.** Summary of Survey Responses (N=260)

**Questions** & *Responses*		

**What is your age?** Mean (median)	42.9 (41)	

	**N**	**%**

**Gender** (N=260)		
*Female*	198	76.2
*Male*	61	23.5
*Prefer not to answer*	1	0.4
*Non-binary*	0	0

**Educational Degree** (N=257)		
*PhD*	117	45.5
*Masters*	63	24.5
*Practice Doctorate (MD PharmD, JD)*	39	15.2
*Bachelors*	37	14.4
*Associates*	1	0.4

**Professional Degree** (N=76)		
*Medical Doctor/Doctor of Osteopathy*	42	55.3
*Public Health*	18	23.7
*Registered Nurse or Advanced Practice Nurse*	9	11.8
*Dietician/Social Worker/Pharmacist*	7	9.2

**Faculty Investigator Role** (N=134)		
*Assistant Professor*	52	38.8
*Associate Professor*	34	25.3
*Professor*	31	23.1
*Instructor/Senior Instructor*	14	10.4
*Other*	3	2.2

**Research Role** (N=260)		
*Faculty investigator*	135	51.9
*Research clinical staff*	82	31.5
*Research administration*	20	7.7
*Non-faculty investigator*	19	7.3
*Research support staff*	4	1.5

**CCTSI Partner Site** (N=260)		
*University of Colorado Anschutz Medical Campus*	203	78.1
*Children’s Hospital Colorado*	67	25.8
*University of Colorado Hospital*	24	9.2
*University of Colorado Boulder*	19	7.3
*University of Colorado Denver*	15	5.8
*Colorado State University*	10	3.8
*Rocky Mountain Regional VA Medical Center*	7	2.7
*Denver Health*	5	1.9
*Kaiser Foundation Research Institute*	5	1.9
*National Jewish Hospital*	2	0.8

**Prior to the remote work recommendation for COVID-19, how many days a week did you work from home?** (N=260)		
*0 days*	133	51.1
*1 day*	69	26.5
*2 days*	30	11.5
*3 days*	9	3.5
*4 days*	5	1.9
*5 days*	9	3.5
*6 days - 7 days (Monday-Friday plus some weekends)*	5	1.9

**To what extent does remote work during COVID-19 interfere with your ability to conduct your research activities?** (N=260)		
*Does not interfere*	62	23.8
*Interferes somewhat*	113	43.5
*Interferes to a great extent*	85	32.7

**Key:** CCTSI: Colorado Clinical and Translational Science Institute
